# Sparse Space Shift Keying Modulation with Enhanced Constellation Mapping

**DOI:** 10.3390/s22155895

**Published:** 2022-08-07

**Authors:** Tiebin Wang, Kaiyuan Huang, Min Liu, Ranran He

**Affiliations:** 1 Information and Computer Engineering College, Northeast Forestry University, Harbin 150006, China; 2 The National Key Laboratory of Science and Technology on Communications, University of Electronic Science and Technology of China, Chengdu 610056, China

**Keywords:** multiple input multiple output (MIMO), space shift keying (SSK), maximum-likelihood (ML)

## Abstract

For reducing the switching frequency between the radio frequency (RF) chain and transmit antennas, a class of new sparse space shift keying modulation (SSSK) schemes are presented. This new class is proposed to simplify hardware implementation, through carefully designing the spatial constellation mapping pattern. Specifically, different from traditional space shift keying modulation (SSK), the proposed SSSK scheme utilizes more time slots to construct a joint design of time and spatial domain SSK modulation, while maintaining the special structure of single RF chain. Since part of the multi-dimension constellations of SSSK concentrate the energy in less time slots, the RF-switching frequency is effectively reduced due to the sparsity introduced in the time domain. Furthermore, through theoretical analysis, we obtain the closed-form expression of the bit error probability for the SSSK scheme, and demonstrate that slight performance gain can be achieved compared to traditional SSK with reduced implementation cost. Moreover, we integrate transmit antenna selection (TAS) to achieve considerable performance gain. Finally, simulation results confirm the effectiveness of the proposed SSSK scheme compared to its traditional counterpart.

## 1. Introduction

The concept of spatial modulation (SM) [1], characterized by the principle of single radio frequency (RF) multiple-input multiple-output (MIMO) design [2,3,4], has attracted considerable attention in research as summarized in [5,6] in order to simplify the implementation of MIMO systems. This is performed by utilizing the index of the activated transmit antenna for information modulation along with traditional digital modulation. Meanwhile, the transmission performance of SM-MIMO was demonstrated to be comparable to traditional MIMO techniques as a possible development direction for future wireless communications toward different applications [7,8], and the unique structure of SM was also suggested to adapt orthogonal frequency division multiplexing (OFDM) [9], massive MIMO [10], high-frequency transmission [11], intelligent surface [12] and wireless security [13]. Following the basic idea of SM, toward a low-cost hardware implementation, space shift keying modulation (SSK) [14] offers an extremely simplified MIMO structure, by inheriting the method of the antenna index modulation process in SM while abandoning the traditional digital modulation. In a nutshell, SSK benefits from a simple RF-switching process at the transmitter, which makes it feasible for scenarios with low-cost devices such as Internet of Things (IoT) [15,16,17].

Meanwhile, with the idea of the fifth generation (5G) becoming reality, the upcoming sixth generation (6G) [18] has focused on even enhanced transmission rate by exploring the use of high-frequency bands as terahertz [19]. Due to the expansive implementation cost on this band, more efficient transmission technologies such as space modulation have been suggested [20] to further reduce the implementation cost. Therefore, the above-mentioned SSK technique has the potential to offer a low-cost MIMO implementation toward 6G wireless communications.

Although the basic idea of SSK focuses on the most efficient implementation of modulation for MIMO, a new challenge lies in that the unaffordable RF-switching frequency becomes a bottleneck for information transmission, which remains an unsolved problem as increasing the implementation cost. To alleviate this issue, a class of offset SSK and SM schemes were developed in [21,22] for reducing the RF-switching frequency. However, the above solution assumes perfect channel state information (CSI) available at the transmitter, which may be not practical in many transmission scenarios. Therefore, reducing the RF-switching frequency without the aid of CSI for traditional SSK modulation remains an attractive challenge. On the other hand, for SSK, the constellation optimization becomes particularly challenging, due to its extremely simplified structure. For example, Hamming-code-aided constellation design was proposed in [23], toward better transmission performance at the cost of the increase in RF chains. Furthermore, extended SSK (ESSK) has been proposed in [24] where the number of active antennas is variable with high spectral efficiency. Furthermore, the performance of ESSK scheme using different detection strategies was evaluated in [24]. To fully exploit the spatial domain to transmit information, Fang, S. et al. [25] proposed a layered space shift keying (LSSK) modulation scheme to further improve the spectrum efficiency which employs a layered architecture of SSK systems. Therefore, on the limitation of utilizing the magnitude of the transmit signal while maintaining the single-RF structure, the constellation optimization for SSK has remained an open challenge. Therefore, of prime concern in this paper is to offer a new constellation mapping method with reduced RF-switching frequency and enhanced system performance.

Against the above background, the major contribution of this paper lies in that, a class of sparse SSK schemes are proposed, characterized by carefully designing and optimizing the constellation in both the spatial and time domain. Meanwhile, with regard to effectively reducing the switching frequency between the RF chain and multiple antennas, part of the multi-dimension constellations of SSSK concentrate the energy of multiple time slots and hence the RF chain does not switch in this duration, while strictly maintaining the single-RF structure of original SSK. Furthermore, a closed-form expression of the union bound on bit error probability for the proposed scheme is also derived by theoretical analysis, in order to demonstrate its slightly improved bit-error rate (BER) performance over original SSK. Finally, in order to further enhance the performance by increasing the transmit diversity, the concept of transmit antenna selection (TAS) is integrated. Specifically, different TAS criteria are firstly compared in terms of transmission performance and computational complexity, then a class of low-complexity TAS algorithms are designed to strike a balanced tradeoff between performance and complexity.

The remainder of this paper is organized as follows. The conventional SSK is reviewed and then the sparse SSK is presented in Section 2, while the comparison of RF-switching frequency is also presented. Section 3 presents the closed-form expression for the union bound on bit error probability of SSSK. In Section 4, transmit antenna selection (TAS) is utilized in sparse SSK to improve the BER performance. Both theoretical and simulation results as well as discussion are presented in Section 5. In Section 6, the conclusion is given.

**Notation** **1.** *We use ${\left(\cdot \right)}^{T}$ and ${∥\cdot ∥}_{F}$ to denote the transpose and the Frobenius norm of a vector/matrix, respectively. P(·) is taken to mean the probability of an event. Q(·) represents the Q-function as $Q\left(z\right)=\frac{1}{\pi }\int_{0}^{\frac{\pi }{2}}exp\left(-\frac{z^{2}}{2sin^{2}\theta }\right)d\theta$. We use $log_{a}\left(\cdot \right)$ for representing the logarithm with base a. Finally, CN$\left(m,\sigma^{2}\right)$ denotes the complex Gaussian distribution of a random variable having independent Gaussian distributed real and imaginary parts, with mean m and variance $\sigma^{2}/2$.*

## 2. SSSK Modulation

### 2.1. Conventional Space Shift Keying Modulation

Let us consider a generic MIMO system with $N_{t}$ transmit and $N_{r}$ receive antennas. Generally, a random sequence of independent bits b $=[b_{1}\;b_{2}\;\ldots \;b_{K}]$ is generated at the transmitter, where *K* represents the sequence length. For the conventional SSK scheme, the groups of $log_{2}N_{t}$ bits are mapped into a constellation vector x $={\left[x_{1}\;x_{2}\;\ldots \;x_{N_{t}}\right]}^{T}$ with a power constraint of unity. The vector x specifies the activated antenna, during which all the other antennas remain idle, and hence have the form x =[00…1…00], in which the element 1 in the vector is the index of the activated antenna. More explicitly, an example of SSK modulation with a spectral efficiency of 2 bits/s/Hz is given in Table 1.

Then the modulation vector is transmitted over an $N_{r}\times N_{t}$ wireless channel H, thus the received signal can be expressed as Y=Hx+W, where the entries of H are assumed to be i.i.d. complex Gaussian random variables with zero means and unit variances and W is the additive white Gaussian noise (AWGN) with mean zero and variance $\sigma^{2}$.

In general, the structure of original SSK offers a special low-cost implementation at the cost of increasing the switching frequency between the RF chain and transmit antennas. Therefore, a new challenge occurs as the overhead of RF-switching frequency. On the one hand, the RF-switching frequency becomes a new bottleneck to enhance the transmission rate. On the other hand, the increase of the RF-switching frequency also introduces extra cost for hardware implementation.

### 2.2. Proposed Sparse Space Shift Keying Modulation

Similarly, we consider an ($N_{t}\times N_{r}$)-element MIMO system for the design of sparse SSK. In this contribution, the SSSK combines multiple moments, i.e., the power of multiple time slots can be concentrated into the constellation mapping toward lower RF-switching frequency. Therefore, the modulated symbol is not a vector but a matrix. Specifically, the transmission data are mapped by the joint design of the spatial domain and time domain so the index formed by the data to be transmitted selects the combination of the activation time slot and the activated transmit antenna. Furthermore, it can be seen from the analysis and simulation results in the following section that this mapping method has comparable BER performance to the conventional SSK. The difference from the original SSK lies in that, the time slot is also treated as a special resource for index modulation, while making the RF-switching frequency considerably reduced because of the introduction of silent slots.

Specifically, to describe SSSK in a detail, we firstly assume that the number of combined time slots is *N*. In *N* slots, an $N_{t}\times N$ matrix X is transmitted on $N_{t}$ antennas, and the matrix X specifies the activated antenna and time slots, during which all other antennas and time slots remain idle. The number of X and the spectral efficiency are expressed as *l* and *m*, respectively.

Now we introduce the concept of SSSK further via the following example in Table 2 with a transmission efficiency of 2 bits/s/Hz transmission mapping rule. Specifically, in Table 2, the case of four transmit antennas and two time slots is considered. Therefore, the four information bits determine the location of the active antenna and time slot, which shows the transmission mapping rule in the context of N=2, $N_{t}=4$. In Table 2, matrix X has $N_{t}^{2}+N_{t}\times N=4^{2}+4\times 2=24$ states and we choose 16 states from the state collection as the constellation of SSSK. To reduce the RF-switching frequency and enhance the system performance, the states with only one time slot activated have the priority. In general, the four information bits determine the location of the active antenna and time slot. Due to the power constraint, when one time slot is activated, the transmit power on the active antenna is $\sqrt{2}$, and when two time slots are activated, the transmit power on the active antenna is 1.

To generalize the basic idea behind Table 2, an algorithm to design the SSSK scheme is summarized as follows.

**Step 1:** Calculate the number of states available for indexing and choose the constellation of SSSK. It is worth mentioning that when N≥3, X is allowed to concentrate the energy on part of the slots. For example, when N=3, matrix X has $N_{t}^{3}+N_{t}\times N+A_{3}^{2}\times N_{t}^{2}=4^{3}+4\times 3+3\times 2\times 4^{2}=172$ states, and 108 of the collection concentrates the energy, which changes the energy distribution thus reducing the switching frequency.

**Step 2:** Divide the information bits $b=[b_{1}\;b_{2}\;\ldots \;b_{K}]$ into bit streams of length *l*, which are mapped to a constellation matrix X.

**Step 3:** Transmit the corresponding X in the MIMO system.

In traditional SSK systems, the special structure requires frequent switching between the transmit antennas and the RF chain. For example, when $N_{t}=4$, the expectation of RF-switching frequency is $\frac{3}{4}$ as the the probability of the case that the constellations in continuous two time slots are different is $\frac{3}{4}$. However, in practical implementation, the switching frequency between RF and antennas is limited. As X in which the RF chain does not switch is considered first in SSSK, the RF-switching frequency of SSSK is considerably reduced.

Similarly as SSK, the SSSK modulation is transmitted over an $N_{r}\times N_{t}$ wireless channel H, then the received signal can be expressed as Y=HX+W, where the entries of H and W are assumed to be CN(m,1) and CN$\left(m,\sigma^{2}\right)$, respectively.

## 3. Performance Analysis

In this section, we analyze the error performance of the developed SSSK system. A tight upper bound on the average bit error probability (BEP) is given by the well-known union bound [11] as:

(1) $$\begin{array}{cc} P_{b} & \leq E_{H}\left[\frac{1}{l2^{l}}\sum_{i=1}^{2^{l}}\sum_{j=1,j\neq i}^{2^{l}}d\left(X_{i}\to X_{j}\right)P\left(X_{i}\to X_{j}|H\right)\right]\\ \; & =\frac{1}{l2^{l}}\sum_{i=1}^{2^{l}}\sum_{j=1,j\neq i}^{2^{l}}d\left(X_{i}\to X_{j}\right)P\left(X_{i}\to X_{j}\right) \end{array}$$

where $P\left(X_{i}\to X_{j}\right)$ is the pairwise error probability (PEP) of deciding SSSK matrix $X_{j}$ given that the SSSK matrix $X_{i}$ is transmitted, and $d\left(X_{i}\to X_{j}\right)$ is the number of bits in error between the matrices $X_{i}$ and $X_{j}$. We obtain the conditional PEP on channel matrix H as

(2) $$\begin{array}{cc} P\left(X_{i}\to X_{j}\right) & =Q\left(\sqrt{\frac{∥H(X_{i}-X_{j}){∥}^{2}}{2N_{0}}}\right). \end{array}$$

Averaging (21) over the channel matrix H and the unconditional PEP is obtained by using the moment generating function (MGF) approach as

(3) $$P\left(X_{i}\to X_{j}\right)=\frac{1}{\pi }\int_{0}^{\frac{\pi }{2}}{\underset{N}{\underbrace{\left(M_{\gamma_{1}}\left(-\frac{1}{sin^{2}\theta }\right)\right)\ldots \left(M_{\gamma_{N}}\left(-\frac{1}{sin^{2}\theta }\right)\right)}}}^{N_{r}}d\theta ,$$

where n=1,2,…,N, and $M_{\gamma_{n}}$ is an MGF of a random variable $\gamma_{n}$ and can be defined as follows

(4) $$M_{\gamma }\left(s\right)=\int_{0}^{\infty }p_{\gamma }\left(\gamma \right)e^{s\gamma }d\gamma .$$

The MGF of most fading distributions can be obtained by standard Laplacian transformation or numerical integration and common fading distributions such as Rayleigh MGF is expressed as

(5) $$M_{\gamma_{b}}\left(s\right)={\left(1-s{\bar{\gamma }}_{b}\right)}^{-1}.$$

Thus, the unconditional PEP can be calculated under Rayleigh fading as

(6) $$P\left(X_{i}\to X_{j}\right)=\frac{1}{\pi }\int_{0}^{\frac{\pi }{2}}{\underset{N}{\underbrace{\left(1+\frac{{\bar{\gamma }}_{1}}{sin^{2}\theta }\right)\ldots \left(1+\frac{{\bar{\gamma }}_{N}}{sin^{2}\theta }\right)}}}^{-N_{r}}d\theta ,$$

where ${\bar{\gamma }}_{n}$ is average signal-to-noise ratio which can be caculated as

(7) $${\bar{\gamma }}_{n}=\frac{\lambda_{i,j,n}}{4N_{0}}$$

where $N_{0}$ is is the power of noise and $\lambda_{i,j,n}$ are the eigenvalues of the distance matrix $\left(X_{i}-X_{j}\right){\left(X_{i}-X_{j}\right)}^{H}$. Consequently, the union bound of the BER of the SSSK system is expressed as

(8) $$\begin{array}{cc} P_{b} & \leq \frac{1}{l2^{l}\pi }\sum_{i=1}^{2^{l}}\sum_{j=1,j\neq i}^{2^{l}}d\left(X_{i}\to X_{j}\right)\int_{0}^{\frac{\pi }{2}}{\underset{N}{\underbrace{\left(1+\frac{{\bar{\gamma }}_{1}}{sin^{2}\theta }\right)\ldots \left(1+\frac{{\bar{\gamma }}_{N}}{sin^{2}\theta }\right)}}}^{-N_{r}}d\theta . \end{array}$$

## 4. Transmit Antenna Selection

When considering the reduction of the number of RF chains, spatial resources could also be utilized to improve the BER performance rather than spectral efficiency, and an abundance of methods have been proposed, among which transmit antenna selection could achieve considerable performance gain in conventional MIMO systems. Specifically, due to the lack of diversity, TAS is usually suggested to adapt SM and SSK in order to enhance the error performance via extra spatial diversity. Focusing on different aspects of the channel matrix, multiple TAS algorithms have been suggested as the norm-based capacity optimized antenna selection (COAS) and the Euclidean distance(ED)-based Euclidean distance optimized antenna selection (EDAS) [26,27].

The aforementioned two algorithms evaluate the channel matrix with totally different criteria, resulting in different tradeoff between complexity and performance. In this treatise, COAS, EDAS and a compromised TAS algorithm are conceived for SSSK systems, while the complexity will be further quantified.

### 4.1. Capacity Optimized Antenna Selection

The COAS uses the simultaneous SNR to evaluate the quality of the selected antenna set. Due to the feature of additive white Gaussian noise, COAS focuses on the Frobenius norm of the selected *q* columns in the channel matrix $H\in C^{N_{r}\times N_{t}}$, which could be expressed as

(9) $$\hat{p}=\arg\max_{p\in S}{∥H_{p}∥}_{F},$$

where p and $\hat{p}$ denote the indices of a certain selection and optimal selection, respectively. S is the index set having Ntq elements. The COAS does not utilize the prior information of the code book at the transmitter, which reduces the complexity compared to ED-based algorithms at the cost of performance gain limitation.

### 4.2. EDAS

Compared to COAS, the EDAS algorithm adopts a different criterion with much increased complexity to select the antennas. In EDAS, the Euclidean distance between transmit vectors which are distorted by the partial channel matrix $H_{p}\in C^{N_{r}\times q}$, and selected from the whole matrix H, is calculated and evaluated, and the minimum distance in $H_{p}$ is then used to select the final antenna set. Therefore, at the cost of high complexity for exhaustive research, the optimal set has the maximum minimum distance. The whole algorithm could be thus depicted as

(10) $$\hat{p}=\arg\max_{p\in S}\left\{\min_{x_{i},x_{j}\in X,i\neq j}{∥H_{p}\left(x_{i}-x_{j}\right)∥}_{F}^{2}\right\},$$

where p and $\hat{p}$ are the indices of a certain selection and the optimal selection, respectively. X is the transmitted vector and x is an element in X. As the Euclidean distance is calculated in each selection and the constellation is traversed, EDAS demonstrates the most excellent performance, with tremendous computational complexity compared to norm-based algorithms.

### 4.3. Compromised TAS Algorithm

In order to balance the performance and complexity for adapting the SSSK scheme, a compromised method of antenna selection, taking both performance and computational complexity into account, is conceived to combine the above-mentioned two criteria. Specifically, a primary selection could be performed with low-complexity norm-based algorithms as COAS, to alleviate the computational complexity, and a complicated, near optimal antenna selection could be performed afterwards to acquire better BER performance. Algorithm 1 could be depicted as follows.
**Algorithm 1** Compromised TAS algorithm.**Input:** X, H, X, $q_{t}$, Initial $H_{t}$=*⌀*, Initial ${\hat{p}}_{t}$=*⌀* **Output:** Selected antenna index set $\hat{p}$ 1:Get ${\hat{p}}_{t}$ using Equation (9) with parameters H and $q_{t}$;2:$H_{t}=H_{\hat{p}}$;3:Get $\hat{p}$ using Equation (10) with parameters $H_{t}$, X and *q*;4:**return** $\hat{p}$.

The parameters $q_{t}$ and $H_{t}$ denote the number of antennas and the temporary channel matrix selected by COAS, respectively. The combination of COAS and EDAS constitutes a balanced trade-off between complexity and performance. As $q_{t}$ increases, better performance could be attained at the cost of complexity, and it is reduced to EDAS when $q_{t}=N_{t}$.

### 4.4. Complexity Analysis

The computational complexity is measured by the real number float operations per second (flops), including complex addition and multiplication. For given complex matrices $A\in C^{a\times b}$, $B\in C^{b\times c}$, $c\in C^{b\times 1}$ and $d\in C^{b\times 1}$, the complexity of c+d, 〈c,d〉, AB and ${\left||A|\right|}_{F}^{2}$ is quantified by 2b flops, 4b−1 flops, 8abc−2ac flops, and 4ab−1 flops, respectively, [28].

Assume an SSSK system with $N_{t}$ transmit antennas, $N_{r}$ receive antennas, M(M<q) time slots and a spectral efficiency *L*, while *q* columns of channel matrix are selected. For convenience, define Nb=Ntq. In COAS, computing the norm of a selected antenna set costs $4N_{r}q-1$ flops. Since there are $N_{b}$ antenna sets to be computed, the overall complexity of COAS is $N_{b}\left(4N_{r}q-1\right)$ flops.

With regard to EDAS, the problem becomes different from that in conventional MIMO systems for the sparsity of SSSK transmit vectors. For any *i* and *j*, $x_{i}-x_{j}$ generates at most 2M non-zero rows, so that the computational complexity of ${∥H_{p}\left(x_{i}-x_{j}\right)∥}_{F}^{2}$ is $2q+16N_{r}M+2N_{r}-1$ flops for a certain *i* and *j*, and the overall complexity is Nb2ML2(2q+16NrM+2Nr−1) flops.

For the compromised algorithm, the TAS process could be divided into two steps, as the primary COAS and EDAS, and the computational complexity is strongly correlated with the temporarily selected antenna number $q_{t}$. From the calculation above, the complexities of COAS and EDAS are Ntqt(4Nrqt−1) and qtq2ML2(2q+16NrM+2Nr−1), respectively. Then the overall computational complexity is Ntqt(4Nrqt−1)+qtq2ML2(2q+16NrM+2Nr−1).

## 5. Simulation and Discussion

In this section, the comparison of RF-switching frequency and a range of numerical BER performance simulation results of the sparse SSK and conventional SSK are presented and compared with different numbers of transmit antennas and receive antennas. In all the simulation results, unless otherwise specified, the Rayleigh fading channel is considered while perfect channel state information is assumed. When channel estimation is not ideal [29,30], the variance of the Gaussian estimation error decreases as the SNR of the data symbols increases, i.e., $\sigma_{e}^{2}={\mathrm{SNR}}^{-1}$. We employ maximal likelihood (ML) detection for both SSK and SSSK schemes. Let $N_{t}$ and $N_{r}$ be the numbers of the transmit and receive antennas, respectively, *N* be the number of combined time slots for SSSK modulation. Moreover, the theoretical curve is given in each figure. As shown in the figures, the upper bound derived becomes very tight upon increasing the SNR values for both SSSK and SSK which is helpful for verifying the correctness of the simulation results. The details for both will be given as follows.

Firstly, to demonstrate the advantages of SSSK in detail, a comparison of the RF-switching frequency between SSK and SSSK is shown in Table 3. We use $E_{SSK}$ and $E_{SSSK}$ to denote expectation of RF-switching frequency of SSK and SSSK, respectively, with the identical spectral efficiency.

**Figure 1:**
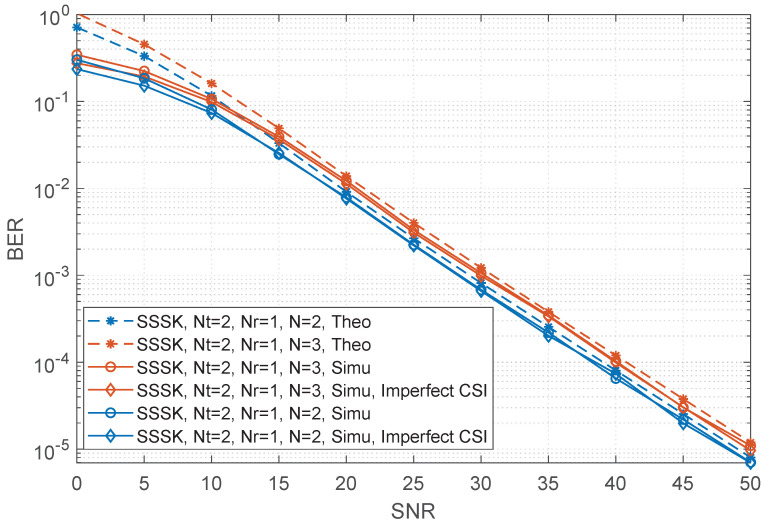
Performance comparisons between the sparse SSK for different values of *N* and the conventional SSK having $N_{t}=2,N_{r}=1$, where the spectral efficiency is equal to 1.5 bits/s/Hz for N=2 and 1.333 bits/s/Hz for N=3.

Subsequently, we present the BER performance curves of the SSSK and SSK modulation with $N_{t}=2,N_{r}=1$ in Figure 1. More specifically, the theoretical and simulation performances of SSSK for N=2 at a spectral efficiency of 1.5 bits/s/Hz and N=3 at a spectral efficiency of 1.33 bits/s/Hz are shown. When SNR increases, the simulation performances gradually approach the theoretical upper bound, which means our theoretical analysis is correct for bounding the performance of proposed SSSK. In general, in the case of $N_{t}=2$, the spectral efficiency of SSSK is higher than that of the traditional SSK. Meanwhile, as seen in Table 3, the RF-switching frequency of SSSK is effectively reduced compared to SSK.

**Figure 2:**
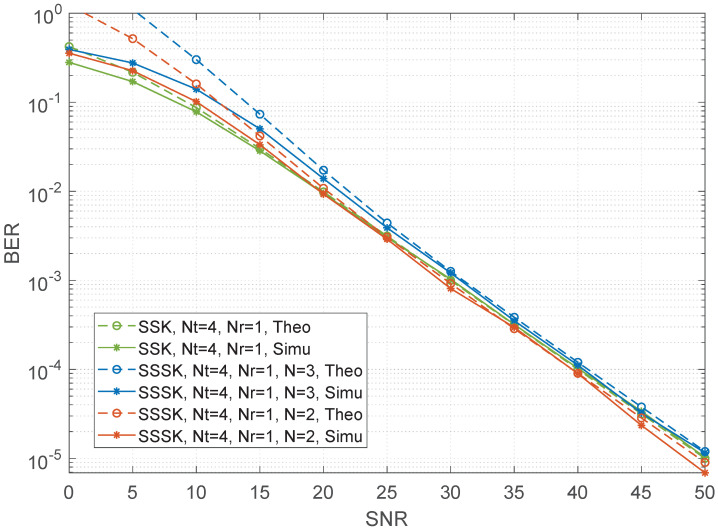
Performance comparisons between the sparse SSK for different values of *N* and conventional SSK having $N_{t}=4$ and $N_{r}=1$ at 2 bits/s/Hz.

**Figure 3:**
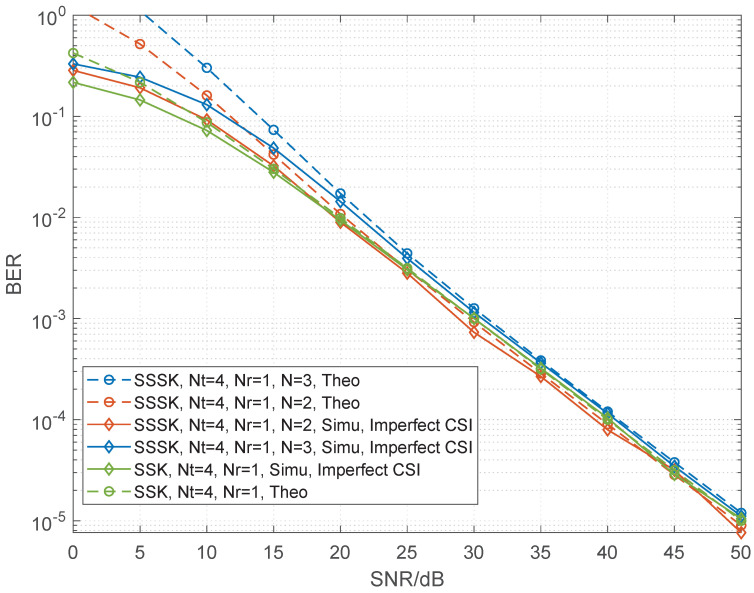
Performance comparisons between the sparse SSK for different values of *N* and conventional SSK having $N_{t}=4$ and $N_{r}=1$ at 2 bits/s/Hz with Gaussian estimation error.

**Figure 4:**
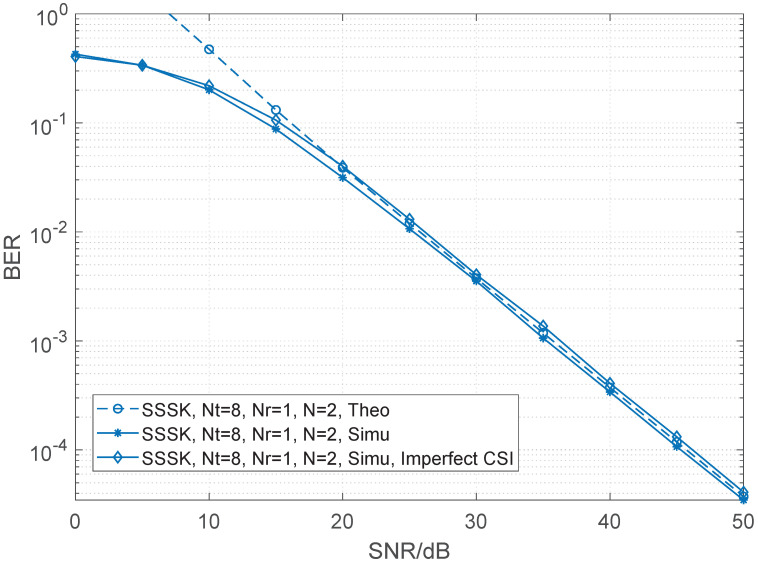
Performance comparisons between the sparse SSK for N=2 and conventional SSK having $N_{t}=8$ and $N_{r}=1$ at 2 bits/s/Hz.

Figure 2 and Figure 3 compare the BER performance of SSSK and conventional SSK having $N_{t}=4$ and $N_{r}=1$ at a spectral efficiency of 2 bits/s/Hz. The figures provide the following observations. Firstly, the BER performance of SSSK improves as *N* decreases under the same antenna configuration. Secondly, when N=2, theoretical and simulation results both show that the BER performance and of SSSK is better than that of the conventional SSK with lower RF-switching frequency. However, as the *N* increases, the advantage of SSSK is gradually reduced. Moreover, we give the results in the case of $N_{t}=8$, $N_{r}=1$ shown in Figure 4. We show the analysis results match the simulation results to demonstrate the correctness of the derived performance.

The above-mentioned antenna selection schemes are validated in SSSK systems, and SSSK without TAS is also simulated as a reference. The total transmit power is set to be unit per time slot. Assume perfect channel state information is available at the receiver and the result of TAS is conveyed to the transmitter through the feedback channel.

**Figure 5:**
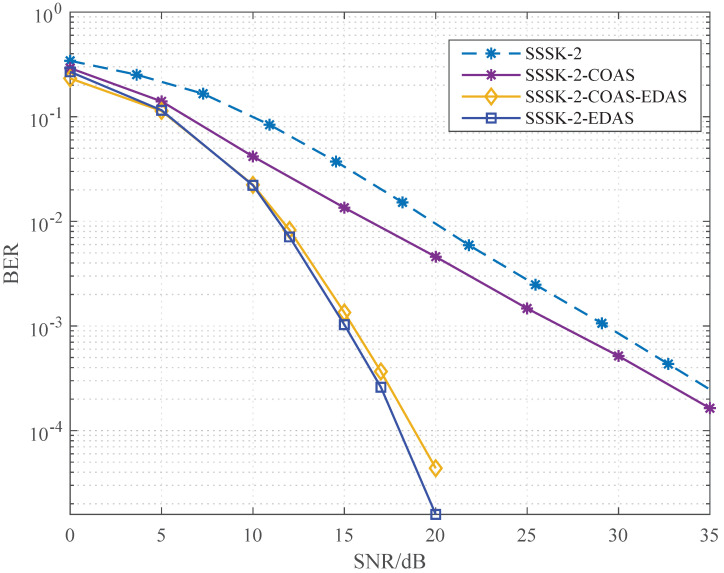
BER performance of SSSK with $N_{t}=8,q=4,N_{r}=1,q_{t}=6$ and *L* = 2 bit/Hz.

Figure 5 shows the BER performance of SSSK with multiple TAS algorithms, in the context of $N_{t}=8,q=4$ and $N_{r}=1$. The SSSK system combines two time slots and the first-stage TAS parameter $q_{t}$ in the compromised TAS algorithm is six. It can be observed from the figures that in the SSSK system COAS could attain a performance gain of 2dB at a BER of $10^{-2}$. The SSSK system obtains excellent performance with ED-based algorithms (EDAS and the compromised TAS algorithm), however, the gap between the compromised algorithm and EDAS becomes quite thin in the SSSK system, implying that the high-complexity EDAS could be replaced by a simplified version with a moderate performance loss.

**Figure 6:**
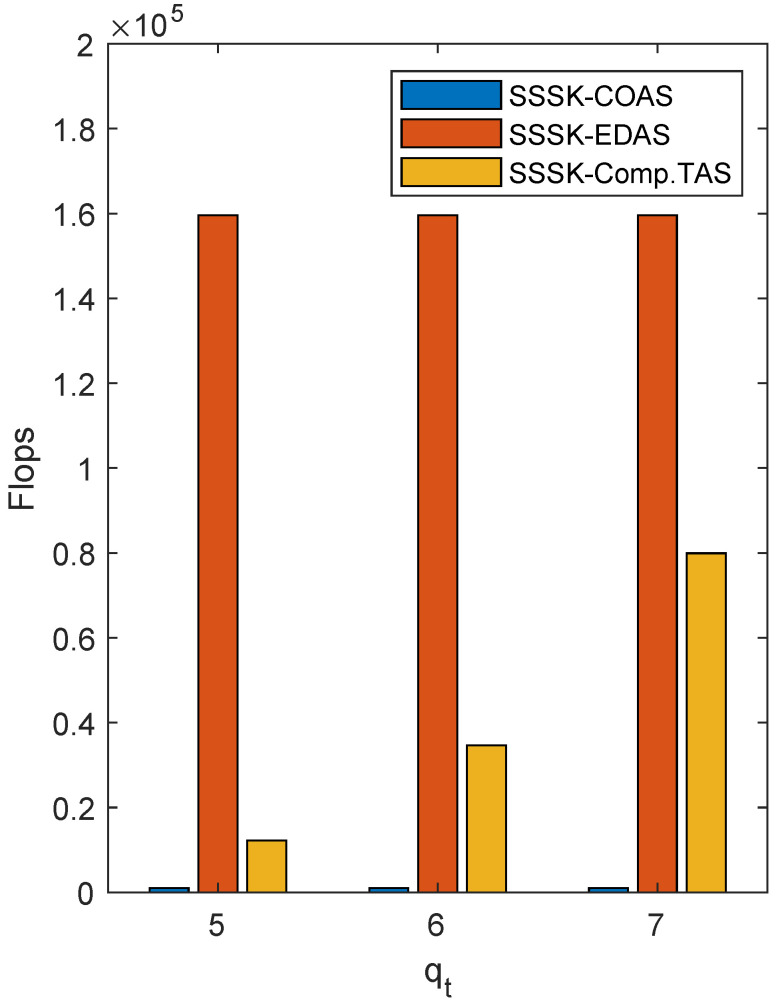
Complexity of SSSK with multiple TAS algorithms with $N_{t}=8,q=4,N_{r}=1$ and *L* = 2 bit/Hz.

The complexity of the aforementioned TAS algorithms is also illustrated in Figure 6 with varied $q_{t}$, indicating that the compromised TAS algorithm could reduce the computational complexity of EDAS effectively. The SSSK system with EDAS possesses highest complexity due to the exhausive search of the maximum minimum Euclidean distance between the transmitted symbols to provide an optimal antenna selection, while the complexity of the system with COAS is much lower, with limited performance gain. The compromised algorithm, however, reduces the complexity of EDAS efficiently by pre-selecting a $q_{t}$ sized antenna set using the simpler COAS algorithm, with a moderate performance degradation compared to EDAS. The complexity of the compromised algorithm increases as $q_{t}$ increases, and when $q_{t}=N_{t}$, the algorithm is increased into EDAS.

In general, through the above-mentioned simulation works, we demonstrate the following issues for the developed SSSK scheme. Firstly, SSSK has the unique advantage of reduced RF-switching frequency without the CSI at the transmitter, while offering slightly improved BER performance compared to its traditional counterpart as SSK. Secondly, the TAS is proved to be effective at combining SSSK for reaping its spatial diversity advantage offered. Lastly, the proposed low-complexity TAS-SSSK is capable of striking a balanced trade-off between performance and complexity.

## 6. Conclusions

In this paper, we introduce a class of sparse SSK modulation techniques toward low-cost implementation of MIMO in terms of reduced RF-switching frequency, characterized by the construction of a joint index modulation in the space and time domains. Furthermore, a theoretical upper bound is achieved through theoretical analysis, to further quantify the performance advantage compared to traditional SSK. In addition, for further improvement of the BER performance through spatial diversity enhancement, the transmit antenna selection is considered while several detailed algorithms are compared. We finally conclude that the developed low-complexity TAS algorithm can strike a balanced trade-off between BER performance and computational complexity.

## Figures and Tables

**Table 1:** Example of the SSK mapper rule.

Input Bits	Antenna Index	Transmission Vector
00	1	${\left[1\;0\;0\;0\right]}^{T}$
01	2	${\left[0\;1\;0\;0\right]}^{T}$
10	3	${\left[0\;0\;1\;0\right]}^{T}$
11	4	${\left[0\;0\;0\;1\right]}^{T}$

**Table 2:** Example of the SSSK mapper rule.

Input Bits	Index	Transmission Vector	Input Bits	Index	Transmission Vector
0000	(1,1)	${\left[\begin{array}{cccc} \sqrt{2} & 0 & 0 & 0\\ 0 & 0 & 0 & 0 \end{array}\right]}^{T}$	1000	(1,1)(1,2)	${\left[\begin{array}{cccc} 1 & 0 & 0 & 0\\ 1 & 0 & 0 & 0 \end{array}\right]}^{T}$
0001	(2,1)	${\left[\begin{array}{cccc} 0 & \sqrt{2} & 0 & 0\\ 0 & 0 & 0 & 0 \end{array}\right]}^{T}$	1001	(1,1)(2,2)	${\left[\begin{array}{cccc} 1 & 0 & 0 & 0\\ 0 & 1 & 0 & 0 \end{array}\right]}^{T}$
0010	(3,1)	${\left[\begin{array}{cccc} 0 & 0 & \sqrt{2} & 0\\ 0 & 0 & 0 & 0 \end{array}\right]}^{T}$	1010	(1,1)(3,2)	${\left[\begin{array}{cccc} 1 & 0 & 0 & 0\\ 0 & 0 & 1 & 0 \end{array}\right]}^{T}$
0011	(4,1)	${\left[\begin{array}{cccc} 0 & 0 & 0 & \sqrt{2}\\ 0 & 0 & 0 & 0 \end{array}\right]}^{T}$	1011	(1,1)(4,2)	${\left[\begin{array}{cccc} 1 & 0 & 0 & 0\\ 0 & 0 & 0 & 1 \end{array}\right]}^{T}$
0100	(1,2)	${\left[\begin{array}{cccc} 0 & 0 & 0 & 0\\ \sqrt{2} & 0 & 0 & 0 \end{array}\right]}^{T}$	1100	(2,1)(1,2)	${\left[\begin{array}{cccc} 0 & 1 & 0 & 0\\ 1 & 0 & 0 & 0 \end{array}\right]}^{T}$
0101	(2,2)	${\left[\begin{array}{cccc} 0 & 0 & 0 & 0\\ 0 & \sqrt{2} & 0 & 0 \end{array}\right]}^{T}$	1101	(2,1)(2,2)	${\left[\begin{array}{cccc} 0 & 1 & 0 & 0\\ 0 & 1 & 0 & 0 \end{array}\right]}^{T}$
0110	(3,2)	${\left[\begin{array}{cccc} 0 & 0 & 0 & 0\\ 0 & 0 & \sqrt{2} & 0 \end{array}\right]}^{T}$	1110	(2,1)(3,2)	${\left[\begin{array}{cccc} 0 & 1 & 0 & 0\\ 0 & 0 & 1 & 0 \end{array}\right]}^{T}$
0111	(4,2)	${\left[\begin{array}{cccc} 0 & 0 & 0 & 0\\ 0 & 0 & 0 & \sqrt{2} \end{array}\right]}^{T}$	1111	(2,1)(4,2)	${\left[\begin{array}{cccc} 0 & 1 & 0 & 0\\ 0 & 0 & 0 & 1 \end{array}\right]}^{T}$

**Table 3:** Comparison of RF-switching frequency.

N	$N_{t}$	$E_{\mathrm{SSK}}$	$E_{\mathrm{SSSK}}$	$E_{\mathrm{SSSK}}/E_{\mathrm{SSK}}$
2	2	0.250	0.5	50%
2	4	0.5547	0.75	73.96%
3	4	0.6745	0.75	89.93%

## Data Availability

Not applicable.

## References

[1] Mesleh R.Y., Haas H., Sinanovic S., Ahn C.W., Yun S. (2008). Spatial modulation. IEEE Trans. Veh. Technol..

[2] Yang P., Xiao Y., Guan Y.L., Hari K., Chockalingam A., Sugiura S., Haas H., Di Renzo M., Masouros C., Liu Z. (2016). Single-carrier SM-MIMO: A promising design for broadband large-scale antenna systems. IEEE Commun. Surv. Tutor..

[3] Li Q., Wen M., Di Renzo M. (2021). Single-RF MIMO: From spatial modulation to metasurface-based modulation. IEEE Wirel. Commun..

[4] Han Z., Zhang Y., Shen S., Li Y., Chiu C.Y., Murch R. (2020). Characteristic mode analysis of ESPAR for single-RF MIMO systems. IEEE Trans. Wirel. Commun..

[5] Yang P., Di Renzo M., Xiao Y., Li S., Hanzo L. (2014). Design guidelines for spatial modulation. IEEE Commun. Surv. Tutor..

[6] Basar E., Wen M., Mesleh R., Di Renzo M., Xiao Y., Haas H. (2017). Index modulation techniques for next-generation wireless networks. IEEE Access.

[7] Xiao Y., Xiao L., Dan L., Lei X. Spatial modulaiton for 5G MIMO communications. Proceedings of the 2014 19th International Conference on Digital Signal Processing.

[8] Basar E. (2020). Reconfigurable intelligent surface-based index modulation: A new beyond MIMO paradigm for 6G. IEEE Trans. Wirel. Commun..

[9] Xiao Y., Wang S., Dan L., Lei X., Yang P., Xiang W. (2014). OFDM with interleaved subcarrier-index modulation. IEEE Commun. Lett..

[10] Sanila K., Rajamohan N. (2021). Enhanced Transmit-Receive Spatial Modulation for Massive MIMO Systems. IEEE Commun. Lett..

[11] Wang T., Jing W., Song W. (2020). Hybrid prefix OFDM with spatial modulation toward terahertz broadband transmission. Sci. China Inf. Sci..

[12] Ma T., Xiao Y., Lei X., Yang P., Lei X., Dobre O.A. (2020). Large intelligent surface assisted wireless communications with spatial modulation and antenna selection. IEEE J. Sel. Areas Commun..

[13] Luo J., Wang H., Wang F., Wang S. (2020). Secure spatial modulation via radio frequency mirrors. IEEE Trans. Veh. Technol..

[14] Jeganathan J., Ghrayeb A., Szczecinski L., Ceron A. (2009). Space shift keying modulation for MIMO channels. IEEE Trans. Wirel. Commun..

[15] Huang Z., Peng Y., Li J., Tong F., Zhu K., Peng L. (2021). Secrecy Enhancing of SSK Systems for IoT Applications in Smart Cities. IEEE Internet Things J..

[16] Mondal A., Hanif M., Nguyen H.H. (2022). SSK-ICS LoRa: A LoRa-Based Modulation Scheme With Constant Envelope and Enhanced Data Rate. IEEE Commun. Lett..

[17] Hanif M., Nguyen H.H. (2020). Slope-shift keying LoRa-based modulation. IEEE Internet Things J..

[18] Yang P., Xiao Y., Xiao M., Li S. (2019). 6G wireless communications: Vision and potential techniques. IEEE Netw..

[19] Chen H., Sarieddeen H., Ballal T., Wymeersch H., Alouini M.S., Al-Naffouri T.Y. (2022). A tutorial on terahertz-band localization for 6G communication systems. IEEE Commun. Surv. Tutor..

[20] Sarieddeen H., Alouini M.S., Al-Naffouri T.Y. (2019). Terahertz-band ultra-massive spatial modulation MIMO. IEEE J. Sel. Areas Commun..

[21] Fang S., Zheng K., Xiao Y., Yang Y., Zeng X., Xiao M. (2019). Offset spatial modulation and offset space shift keying: Efficient designs for single-RF MIMO systems. IEEE Trans. Commun..

[22] Dan L., Jiang T., Xiao Y., Xiao M., Fang S. (2021). Design of offset spatial modulation ofdm. IEEE Trans. Commun..

[23] Chang R.Y., Lin S.J., Chung W.H. (2011). New space shift keying modulation with Hamming code-aided constellation design. IEEE Wirel. Commun. Lett..

[24] Mokh A., Hélard M., Crussière M. Extended Space Shift Keying Modulation With Different Receiver Strategies. Proceedings of the 2019 26th International Conference on Telecommunications (ICT).

[25] Fang S., Li L., Hu S., Tang J., Yue Z., Feng G., Pandharipande A. (2016). Layered space shift keying modulation over MIMO channels. IEEE Trans. Veh. Technol..

[26] Rajashekar R., Hari K., Hanzo L. (2013). Antenna selection in spatial modulation systems. IEEE Commun. Lett..

[27] Pillay N., Xu H. (2013). Comments on “Antenna Selection in Spatial Modulation Systems”. IEEE Commun. Lett..

[28] Xiao L., Yang P., Xiao Y., Fan S., Di Renzo M., Xiang W., Li S. (2016). Efficient compressive sensing detectors for generalized spatial modulation systems. IEEE Trans. Veh. Technol..

[29] Neumann D., Wiese T., Utschick W. (2018). Learning the MMSE channel estimator. IEEE Trans. Signal Process..

[30] Liu C., Liu X., Ng D.W.K., Yuan J. (2021). Deep residual learning for channel estimation in intelligent reflecting surface-assisted multi-user communications. IEEE Trans. Wirel. Commun..

